# GeTe-TiC-C Composite Anodes for Li-Ion Storage

**DOI:** 10.3390/ma13194222

**Published:** 2020-09-23

**Authors:** Woo Seob Kim, Thuan Ngoc Vo, Il Tae Kim

**Affiliations:** Department of Chemical and Biological Engineering, Gachon University, Seongnam-si, Gyeonggi-do 13120, Korea; termies@naver.com (W.S.K.); vnthuanbk@gmail.com (T.N.V.)

**Keywords:** germanium telluride, titanium carbide, conductive matrix, anodes, lithium-ion batteries

## Abstract

Germanium boasts a high charge capacity, but it has detrimental effects on battery cycling life, owing to the significant volume expansion that it incurs after repeated recharging. Therefore, the fabrication of Ge composites including other elements is essential to overcome this hurdle. Herein, highly conductive Te is employed to prepare an alloy of germanium telluride (GeTe) with the addition of a highly conductive matrix comprising titanium carbide (TiC) and carbon (C) via high-energy ball milling (HEBM). The final alloy composite, GeTe-TiC-C, is used as a potential anode for lithium-ion cells. The GeTe-TiC-C composites having different combinations of TiC are characterized by electron microscopies and X-ray powder diffraction for structural and morphological analyses, which indicate that GeTe and TiC are evenly spread out in the carbon matrix. The GeTe electrode exhibits an unstable cycling life; however, the addition of higher amounts of TiC in GeTe offers much better electrochemical performance. Specifically, the GeTe-TiC (20%)-C and GeTe-TiC (30%)-C electrodes exhibited excellent reversible cyclability equivalent to 847 and 614 mAh g^−1^ after 400 cycles, respectively. Moreover, at 10 A g^−1^, stable capacity retentions of 78% for GeTe-TiC (20%)-C and 82% for GeTe-TiC (30%)-C were demonstrated. This proves that the developed GeTe-TiC-C anodes are promising for potential applications as anode candidates for high-performance lithium-ion batteries.

## 1. Introduction

In recent times, the number of customers interested in purchasing electric vehicles and electronic devices is steadily increasing, and these products demand long-lasting energy storage systems. Thus, the energy storage market has continuously and significantly grown over the past decades; specifically, batteries and battery-related technologies have become increasingly vital for modern technology [1,2,3]. Lithium-ion batteries (LIBs) have been commonly utilized globally as energy storage systems. Owing to the desirability of long-lasting operation with one charge, the demand for LIBs with higher energy density and more stable performance is steadily increasing. To date, graphite anodes have been applied in LIBs; however, they exhibit a low theoretical capacity (≈372 mAh g^−1^), low tap density (<1 g cm^−3^), and low energy density. Further, safety issues and performance degradation related to Li plating on the graphite surface remain unresolved, which has encouraged the exploration of new anode materials [4,5,6,7,8]. To address the aforementioned disadvantages, various Li-alloying elements such as Sb, Sn, Si, and Ge have been proposed as potential anode materials for replacing graphite [9,10,11,12,13,14,15,16]. Ge exhibits a higher theoretical capacity (1385 mAh g^−1^) than that (≈372 mAh g^−1^) of graphite, an electrical conductivity (2.1 S m^−1^) [10,17,18] higher than Si (1.6 × 10^−3^ S m^−1^), and a lithium diffusivity 400 times faster than Si [19,20,21,22]. Despite these advantages, Ge anodes face a major disadvantage pertaining to poor cycling because of the 300% volume variation during repeated electrochemical process [23,24]. In this regard, effectual strategies are needed to provide structural stability for the composites by generating effective buffer phases and preventing nanoparticle agglomerations [8,23,24,25,26,27,28,29,30]. Among these, titanium carbide (TiC) could be used to fabricate an outstanding structural barrier, with its high conductivity and hardness [31,32]. Due to the specific properties of TiC, various studies on the electrodes with TiC and/or C have been explored, where the addition of TiC to the composite electrodes made it possible to show stable cyclic performances [23,33,34,35,36,37]. In addition, conductive carbon can enhance nanoparticle separation and the conductivity of composites, and it can also stabilize electrochemical performances for LIBs [24,28,38]. Meanwhile, for reducing the large volume expansion of the metallic Ge, intermetallic components that act as a buffering step could be incorporated into composite electrodes. For this purpose, the chalcogens of sulfur (S), selenium (Se), and tellurium (Te) are potential candidates and can be alloyed with Li upon the Li_2_X (X = S, Se, Te) phase formation [39,40,41,42,43,44,45,46,47,48,49,50]. Among them, Te has a reasonable theoretical capacity (420 mAh g^−1^) with a high conductivity (2.0 S cm^−1^). Moreover, Te reacts with Li ions at around 0.9–1.9 V to form Li_2_Te [35,46,48,49,50,51]. Meanwhile, Ge has a different reaction potential (≈0.6 V vs. Li/Li^+^); thus, Ge and Te react independently with lithium ions at separate potentials. Thus, it is expected that the formation of intermetallic germanium telluride (GeTe) complexes in composites will effectively diminish significant volume variations upon cycling, owing to the possession of different redox potentials with Li, leading to good electrochemical performance. In this study, based on the aforementioned scenario, the following strategies were chosen to obtain outstanding electrochemical performance. (1) Hybrid TiC and C matrices were developed. They led to an increase in conductivity, which generated the buffering effect, accommodated the volume change during cycles, and facilitated electron transport as well as Li ion diffusion. (2) The existence of Te with Ge suppressed the abrupt volume change. Based on these strategies, the fabricated GeTe-TiC-C composite electrode is expected to demonstrate superior electrochemical performance.

## 2. Experimental

### 2.1. Preparation of GeTe and GeTe-TiC-C

**Figure 1:**
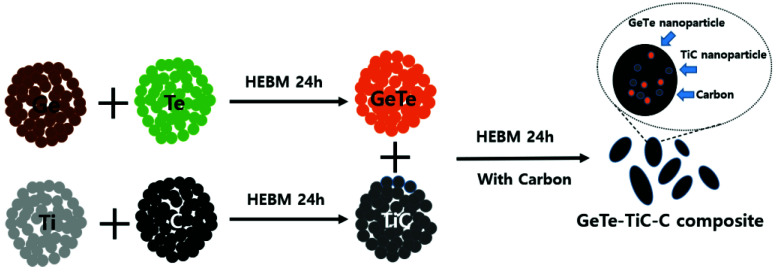
Schematic for the formation of GeTe-TiC-C composite.

To synthesize GeTe and GeTe-TiC-C nanocomposites, a 1:1 molar ratio of Ge (99.999%, ~100 mesh, Alfa Asear, Black freer, MA, USA) and Te (99.99%, ~100 mesh, Aldrich, St. Louis, MO, USA) were mixed, which were sealed in an Ar-filled glove box and high-energetically milled (Pulverisette 5, Fritsch, Markt Einersheim, Germany), with a powder:ZrO_2_ ball ratio of 1:20 for 24 h at a speed of 300 rpm. Similarly, acetylene black (99.9%, Alfa Asear) and Ti (99.99%, ~325 mesh, Alfa Aesar) were mixed with a molar ratio of 1:1 and high-energetically milled for 24 h in the same condition, thus forming the TiC phase. Subsequently, the synthesized GeTe and TiC were mixed with acetylene black and prepared with different wt % ratios (GeTe:TiC:C = 70:10:20, 60:20:20, and 50:30:20) in a container sealed with Ar gas. The prepared powder mixtures were milled again for 24 h at 300 rpm, which generated the final product—GeTe-TiC-C composites. The entire synthesis procedure is depicted in Figure 1.

### 2.2. Material Characterization

The structure analysis of the as-synthesized powder materials was performed in the 2θ range of 20°–60° at a scan speed of 2° min^−1^ using X-ray diffraction (XRD) (D/MAX-2200, Rigaku, Tokyo, Japan) with the software of Smartlab studio 2 at the Smart Materials Research Center for IoT in Gachon University (v4.3.177.0, Tokyo, Japan). Scanning electron microscopy (SEM, Hitachi S-4700, Tokyo, Japan) was utilized in order to examine the morphology of GeTe and GeTe-TiC-C. High-resolution transmission electron microscopy (HRTEM, JEOL 2100, Tokyo, Japan) furnished with energy-dispersive X-ray spectroscopy (EDS) was used for analyzing the forms and compositions of the elements. For obtaining tap density with the unit of g cm^−3^, the active material was put into a small measuring cylinder (10 mL) and tapped for 10 min so that the active material was packed well. Then, the weight of the active material was measured from the difference in weight between the empty measuring cylinder and the one occupied with active material. After measuring the volume of the active material, the tap density was calculated from the obtained volume and weight.

### 2.3. The Measurement of the Electrochemical Properties

To investigate the electrochemical properties, 70 wt % active materials (GeTe or GeTe-TiC-C powder), 15 wt % Super P (conductive carbon black), and 15 wt % poly acrylic acid were homogenized thoroughly in an ethyl alcohol solution and cast in slurry on a flexible copper foil. After casting, they were dried overnight at 70 °C under vacuum condition. The electrodes were punched into a circular shape with a diameter of 12.5 mm; moreover, they possessed 1–1.5 mg of GeTe or GeTe-TiC-C material. The CR2032-type half-cell was fabricated with 1 M LiPF_6_ in ethylene carbonate (EC) and diethyl carbonate (DEC) (1:1 v/v) for use as the electrolyte; the working electrode consisted of GeTe or GeTe-TiC-C, and the counter electrode comprised of Li foil. The galvanostatic tests (WBCS300, WonAtech, Seoul, Korea) were conducted at a current density of 100 mA g^−1^ at 25 °C, and a fast-charging-performance test was performed at various current rates of 0.1, 0.5, 1, 3, 5, and 10 A g^−1^ by considering the weight of the electroactive materials (GeTe or GeTe-TiC-C). Cyclic voltammetry (CV) as well as electrochemical impedance spectroscopy (EIS) analyses were performed using the ZIVE MP1 (WonAtech). The cyclic voltammograms were obtained in the potential range of 0.001–2.5 V versus Li/Li^+^, with a scan rate of 0.1 mV s^−1^, and EIS data were obtained by applying an amplitude of 5 mV over the frequency range of 100 kHz–100 mHz after the 100th cycle.

## 3. Results and Discussion

**Figure 2:**
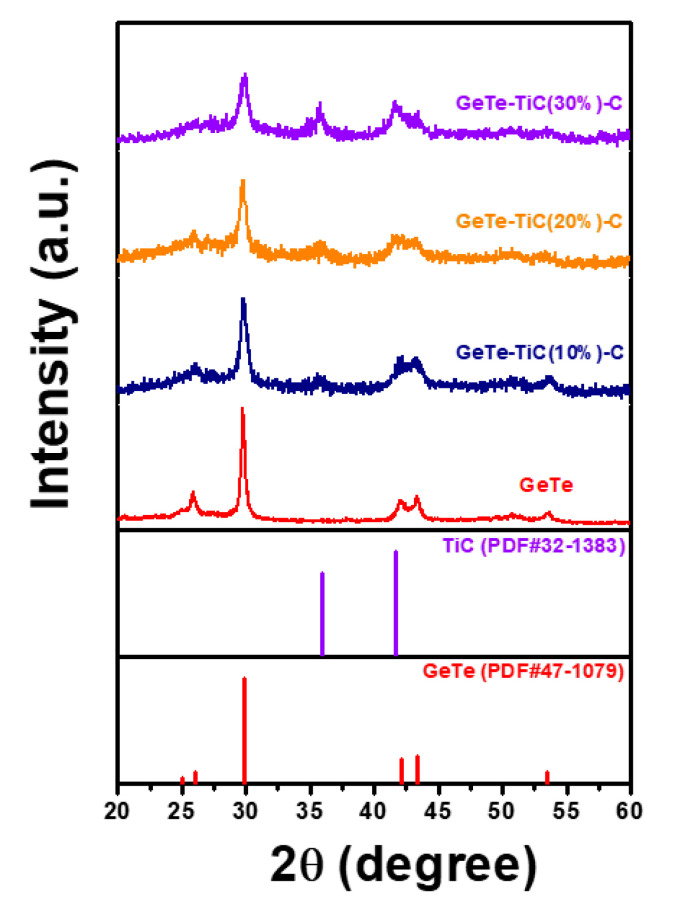
X-ray diffraction (XRD) patterns of GeTe and GeTe-TiC-C composites.

The GeTe and GeTe-TiC-C powder samples are evaluated by XRD, and Figure 2 shows the XRD peaks of the as-prepared composites. To develop the targeted sample (GeTe-TiC-C), Ge/Te, and Ti/C were mechanically milled. Then, acetylene black was added with a fixed weight percent (20%). The XRD peaks corresponding to GeTe (PDF #47-1079) and TiC (PDF #32-1383) coincided well with the reference peaks. The analysis of the GeTe-TiC-C composites with different weight percentages of TiC leads to the observation that when the TiC ratio increases, the intensity of the TiC peak increases, whereas a decrease in the GeTe peaks is observed.

**Figure 3:**
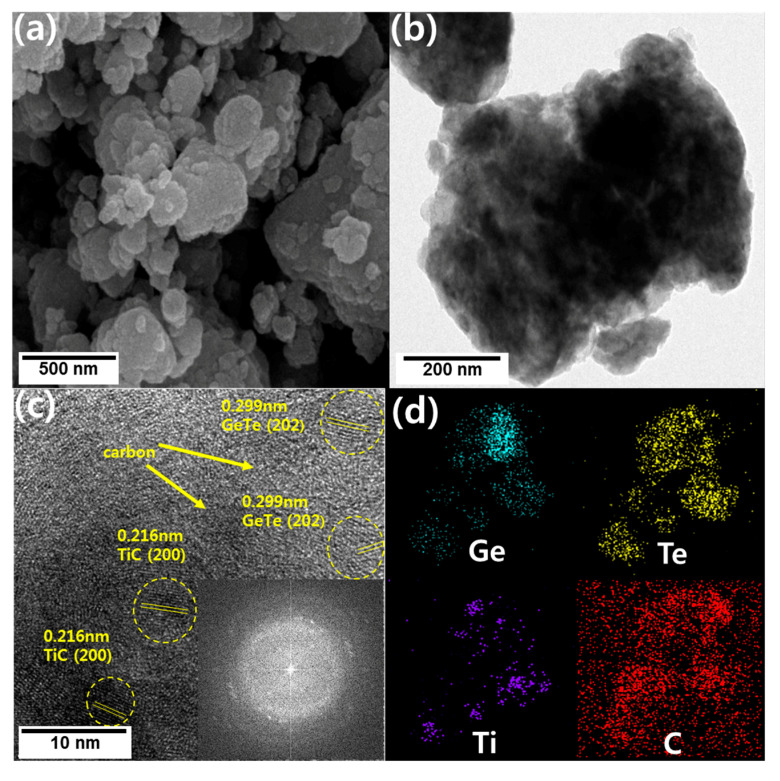
(**a**) Scanning electron microscopy (SEM), (**b**) Transmission electron microscopy (TEM), and (**c**) high-resolution TEM images of GeTe-TiC (20%)-C. (**d**) Energy-dispersive X-ray spectroscopy (EDS) mapping images of GeTe-TiC (20%)-C from Scanning transmission electron microscopy (STEM).

The morphologies of GeTe-TiC (20%)-C samples are shown in Figure 3. From the SEM and TEM images (Figure 3a,b), it is observed that irregular-shaped micro/nanoparticles were aggregated. Moreover, well-defined crystalline structures of GeTe and TiC were detected as evident from the high-resolution morphology images in Figure 3c, where interlayer spacing of 0.299 nm for GeTe (202) and 0.216 nm for TiC (200) crystalline phases were observed. The selected-area electron diffraction patterns also displayed several spots corresponding to the crystalline natures from GeTe and TiC. The amorphous regions could be designated as a part of the carbon matrix. Finally, the EDS-mapped images illustrate that each component including Ge, Te, Ti, and C are well-dispersed in the composite.

**Figure 4:**
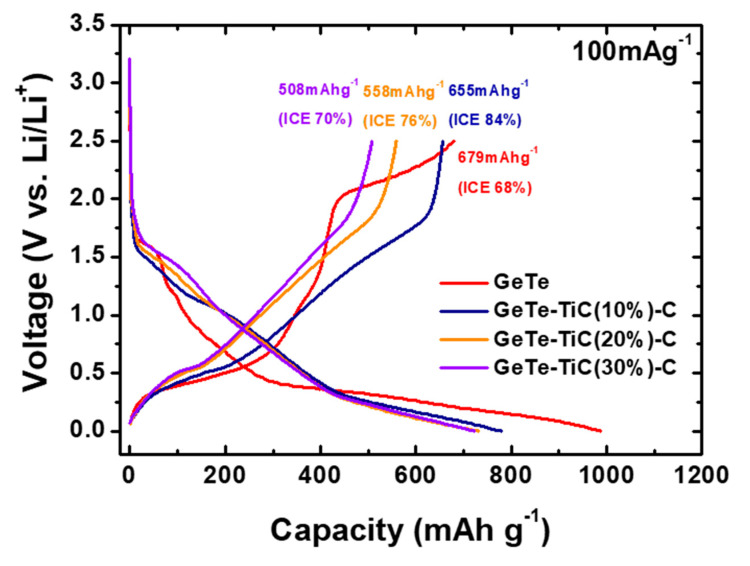
Initial voltage profiles of GeTe and GeTe-TiC-C (TiC: 10, 20 and 30 wt %) electrodes at a constant current density of 100 mA g^−1^.

The initial potential profiles of the as-prepared GeTe and GeTe-TiC-C composite electrodes were analyzed at 100 mA g^−1^, as shown in Figure 4. Additionally, the corresponding electrochemical data are summarized in Table 1. During the first discharge and charge cycle of the GeTe-TiC (10%)-C, GeTe-TiC (20%)-C, and GeTe-TiC (30%)-C electrodes, a sloping plateau region appears at approximately 0.25 V, which is attributed to the electrochemical reaction between Ge and Li ions. Furthermore, the other sloping plateau can be detected from 1.6 to 0.9 V, corresponding to the electrochemical reaction between Te and Li ions. GeTe-TiC-C composites displayed sloping plateaus, unlike GeTe. This is because the crystalline nature starts disappearing, owing to the high TiC content [37,52]. The diffusion of Li ions is prevented in the 1st cycle, resulting in more side reactions and the development of the solid electrolyte interface (SEI) layer. Meanwhile, regarding the electrochemical initial performance, during the first cycle of the GeTe electrode, the discharge and charge capacities were 987 and 679 mAh g^−1^, respectively. The initial capacity loss is usually attributed to the development of SEI layers on the electrode surface. In this case, GeTe electrode displayed exceedingly poor electrochemical performances, with a considerably low initial Coulombic efficiency of 69%. This inferior performance can be attributed to the absence of TiC and carbon, which acted as the buffering agent. In contrast, the composite anodes with the presence of TiC and C exhibited significantly better initial performance. Specifically, during the first cycle, the charge and discharge values of the GeTe-TiC-C electrodes respectively were 655 and 778 mAh g^−1^ for GeTe-TiC (10%)-C, 558 and 729 mAh g^−1^ for GeTe-TiC (20%)-C, and 508 and 722 mAh g^−1^ for GeTe-TiC (30%)-C electrodes, where their initial Coulombic efficiencies were higher than that of the GeTe electrode (refer Table 1). Additionally, the increase in the TiC content reduced the initial charge capacity, resulting from the low activity of TiC with Li ions. Furthermore, the initial Coulombic efficiency also decreased with an increase in TiC content. Although TiC could significantly improve the stability of the composite electrodes, it was found that an excess amount of TiC frequently caused a decline in the initial Coulombic efficiency [35,53]. Nevertheless, after long cycling, the electrodes with TiC content exhibited outstanding capacity retentions (discussed later).

**Figure 5:**
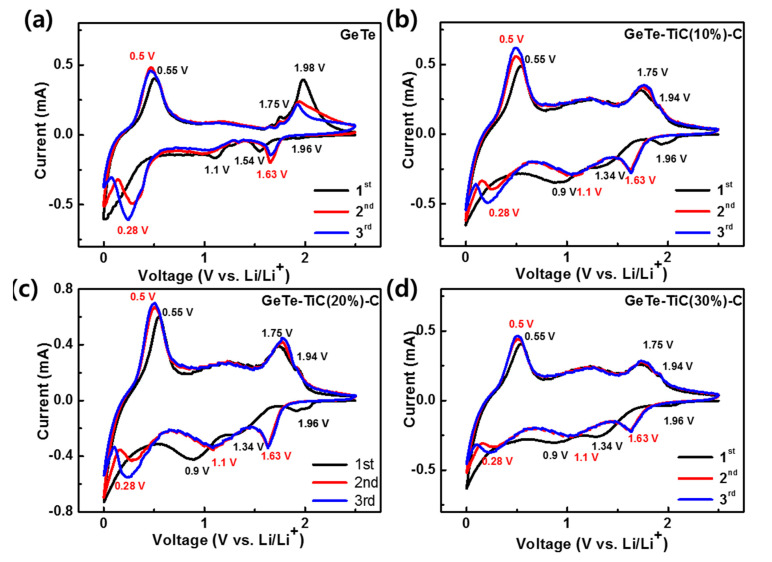
Cyclic voltammograms of (**a**) GeTe, (**b**) GeTe-TiC (10%)-C, (**c**) GeTe-TiC (20%)-C, and (**d**) GeTe-TiC (30%)-C in the three initial cycles at 0.1 mV s^−1^.

The CV test was conducted to further elucidate the electrochemical reactions. Figure 5 shows the initial cyclic voltammograms of GeTe and GeTe-TiC-C electrodes within a voltage range of 0–2.5 V at 0.1 mVs^−1^. The potential plots of GeTe-TiC-C electrodes exhibit the analogous behavior. In the initial lithiation process, the reaction potentials from 1.9 to 0.9 V illustrated the alloy reactions of Te with Li ions to form the Li_2_Te phase $\left(\mathrm{GeTe}\;+2{\mathrm{Li}}^{+}\to {\mathrm{Li}}_{2}\mathrm{Te}\;+\mathrm{Ge}\right)$ [35,51]. In practice, the redox potential of Te strongly relied on morphologies and dispersing matrices [54,55]. Figure 5a disclosed that the lithiation of Te occurred at 1.1 and 1.54 V in the first cycle for the as-prepared GeTe. In fact, stress-related factors frequently influenced the lithiation potential of the chalcogens [44]. Due to the stress caused by the TiC matrix after the embedding process, only one cathodic peak at 1.34 V was observed in the first cycle for the lithiation of Te in GeTe-TiC-C composites. From the second cycle, for all the composite electrodes, the relaxation of Te split the aforementioned cathodic peak (1.34 V) into two peaks at 1.1 V and 1.63 V, which were identical to the lithiation potential of Te in the GeTe electrode (Figure 5). In addition, the cathodic peak at 1.96 V could correspond to an additional SEI formation, which occasionally happened in Te-based electrodes. For example, by embedding ultrafine Te particles in carbon matrices, Liu et al. observed an irreversible cathodic peak at ≈2 V for the 1st cycle [50].

Moreover, a broad peak was observed at approximately 0.25 V on further discharging to a lower voltage, which was ascribed to the formation of the Li_3.75_Ge phase along with the solid electrolyte interphase layer formation on the surface of the electrode material $\left(\mathrm{Ge}\;+3.75{\mathrm{Li}}^{+}\;\to {\mathrm{Li}}_{3.75}\mathrm{Ge}\right)$. In 2015, Ngo et al. suggested that the lithiation of Ge might reveal multiple cathodic peaks, whereas the SEI formation occurred right before the lowest lithiation potential of Ge during the discharging [56]. Accordingly, it is reasonable to attribute the broad cathodic peak at 0.25 V to an overlap of two reactions: a lithiation of Ge and SEI formation. Meanwhile, in the initial delithiation process, the sequential reversible reaction of Li_3.75_Ge to Ge occurred when the potential increased from 0.01 to 0.55 V (${\mathrm{Li}}_{3.75}\mathrm{Ge}\;\to \mathrm{Ge}\;+3.75{\mathrm{Li}}^{+})$ [57]. In the further charged state, two oxidation peaks were observed at 1.75 and 1.98 V for GeTe, and 1.75 and 1.94 V for GeTe-TiC-C electrodes. Both reaction potentials were related to the dealloying reaction of the Li_2_Te phase $\left({\mathrm{Li}}_{2}\mathrm{Te}\;+\mathrm{Ge}\;\to \mathrm{GeTe}\;+2{\mathrm{Li}}^{+}\right)$. In the case of the GeTe electrode, it showed the main peak at 1.98 V with a weak shoulder peak at 1.75 V. On the other hand, GeTe-TiC-C electrodes revealed the main peak at 1.75 V with a weak peak at 1.94 V. This might be because the introduction of TiC-C into GeTe changed the main reaction potentials. In addition, the broad cathodic and anodic peaks at around 1 V were observed, which might be related to the alloying/dealloying reactions of Te and Li as well [35]. The large broadness of the peaks could be due to the gradual (de)alloying reactions; then, main reaction at a redox potential of 1.63/1.75 V occurred. After the first cycle, the reduction peaks are shifted toward a higher potential, while all the oxidation peaks were shifted slightly to a lower voltage, illustrating the reduced electrode polarization during the discharge–charge processes [33,50,57]. During the first discharge process, unstable and wide peaks were observed in all the electrodes, corresponding to the SEI layer formation. The peaks were stabilized from the second and third cycles for GeTe-TiC-C electrodes, which is a result of the reversible electrochemical reactions. The overall reaction mechanism can be summarized as follows.

In the discharge process:

GeTe → Ge + Li_2_Te → Li_3.75_Ge + Li_2_Te

In the charge process:

Li_3.75_Ge + Li_2_Te → Ge +Li_2_Te → GeTe.

**Figure 6:**
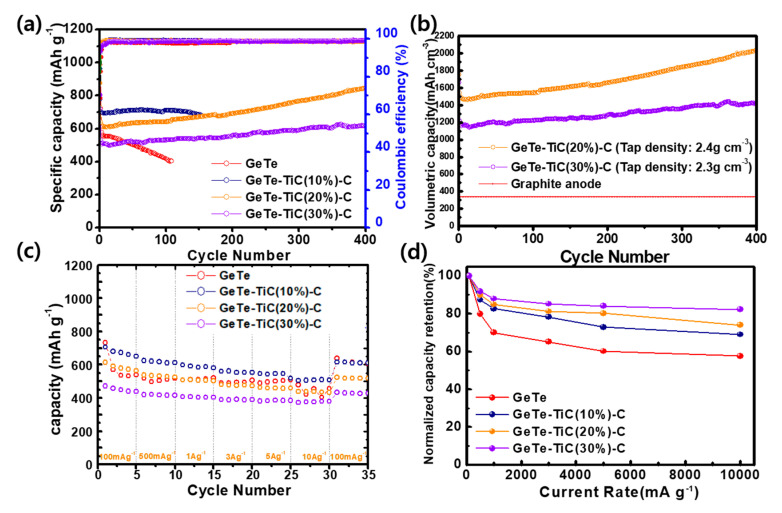
Cycling performance comparison of GeTe and GeTe-TiC-C anodes at 100 mA g^−1^ for (**a**) gravimetric capacity and (**b**) volumetric capacity. (**c**) Rate performance of GeTe and GeTe-TiC-C anodes at different current densities and (**d**) their capacity retentions.

The cyclic performances and the Coulombic efficiency of GeTe and GeTe-TiC-C electrodes at 100 mA g^−1^ are compared in Figure 6a. The GeTe electrode has the highest initial discharge capacity (987 mAh g^−1^); however, the capacity retention after 100 cycles was approximately 58% with respect to the value in the 2nd cycle, which proves its rapid fading capacity. This implies that only GeTe was unable to endure the large volume expansion. The GeTe alloy possesses large volume expansion and critical aggregation issues during the cycling, which could lose the electrical contact with the binder/super P/current collector and cause the pulverization of the electrode [33,35,36]. In contrast, the cyclic performances were much improved by introducing the TiC-C buffering matrix into the GeTe alloy. The GeTe-TiC (10%)-C electrode exhibits a better discharge capacity of 711.4 mAh g^−1^ than those of GeTe-TiC (20%)-C and GeTe-TiC (30%)-C after 100 cycles, resulting from the higher GeTe active material content in the composite compared to others. However, the capacity gradually decreases up to the 156th cycle. This could be attributed to the insufficient amount of TiC in the composite that could not sufficiently prevent the GeTe active particles from the volume expansion during repeated cycling. Composites with higher TiC weight percent, the GeTe-TiC (20%)-C and GeTe-TiC (30%)-C electrodes, displayed discharge capacities of 641.6 and 531.7 mAh g^−1^, respectively, after 100 cycles, which correspond to high capacity retentions of ≈99% with respect to the values in the 2nd discharge capacity. With a further increase in cycles, the electrodes showed a gradual increase in capacity, leading to over 100% capacity retention. The gradual increase in capacity upon long cycling may be attributed to the activation process triggered by the gradual wetting and soaking of the electrolyte into electrodes [33,58,59]. Most importantly, the changes in the capacity plateaus (Figure S1) after some cycles support the reversible establishment and degradation of polymer/gel-like film that serves as a reservoir for the storage of excess Li^+^ ions via a pseudocapacitance-type behavior [33,60]. This could be owing to the electrolytes decomposition and the electrochemical catalytic effect of the formed nanoparticles (NPs) on accelerating or activating the decomposition of certain SEI [61]. The two electrodes with high TiC content (20% and 30%), when conducting a long-lasting cyclic test, exhibited superior cyclic performance even after 400 cycles. When the TiC content was optimized to be 20 wt % (GeTe-TiC (20%)-C), both high-capacity and cyclic stability were realized. In particular, it exhibited a capacity of 847.1 mAh g^−1^ after 400 cycles. This can be attributed to the introduction of an appropriate amount of TiC dispersed uniformly in the matrix, which effectively suppresses the aggregation of active GeTe, increases the conductivity of the entire matrix, reduces the particle size, and mitigates the stresses and strains during the extended cycling [33,35,36]. Moreover, the developed composites possess high tap densities of 2.4 g cm^−3^ for GeTe-TiC (20%)-C and 2.3 g cm^−3^ for GeTe-TiC (30%)-C. Based on these values, the volumetric capacity was obtained by multiplying the specific capacity and tap density. Figure 6b shows the volumetric capacity of GeTe-TiC-C electrodes; for comparison, the theoretical volumetric capacity for graphite is plotted as a line. The GeTe-TiC (20%)-C electrode demonstrated a volumetric capacity of 1657 mAh cm^−1^ after 200 cycles. Furthermore, the GeTe-TiC (30%)-C electrode exhibited a volumetric capacity of 1281 mAh cm^−3^ after the same number of cycles. The volumetric capacity values of GeTe-TiC-C electrodes are three or five times greater than the theoretical volumetric capacity of the graphite anode (372 mAh g^−1^ × 0.9 g cm^−3^ = 335 mAh cm^−3^), indicating that the composite electrodes possess sufficient potential as an alternative. The measured tap density of graphite was consistent with that reported in other study [62]. Table S1 shows a comparison of the cyclic performance of electrodes with TiC content in composite materials. When comparing the developed electrodes with others, the GeTe-TiC-C electrodes demonstrated stable cyclability (Figure 6a,b) and high reversible capacity upon long lasting cycling, which are comparable to other electrodes. Interestingly, we found that the GeTe-TiC-C possessed a long activation time, which hardly happened for other telluride-based materials such as SnTe-TiC-C [35]. Moreover, GeTe-TiC-C composites were overall higher than SnTe-TiC-C composites in reversible capacity. Namely, after 400 cycles, our optimized composite electrode delivered a reversible gravimetric capacity (847 mAh g^−1^) that was twice that of SnTe-based electrode (≈400 mAh g^−1^).

Fast charging with high efficiency has been required by industries in recent years. Figure 6c,d display the results of the rate performance obtained by varying current densities from 0.1 to 10 A g^−1^. The GeTe electrode demonstrated an unstable rate performance, which is similar to the result of cyclability. The addition of TiC and C into composites improved the rate stability of the GeTe-TiC-C electrodes [52,53]. For instance, the charging capacity of the GeTe-TiC (10%)-C electrode at 10 A g^−1^ was 485 mAh g^−1^. It further displayed a slightly higher capacity retention (69%) than the GeTe electrode (57%). When the amount of TiC was applied as 20 wt % and 30 wt % in the composite, the GeTe-TiC (20%)-C and GeTe-TiC (30%)-C electrodes exhibited excellent rate capacity retention corresponding to 78% and 82%, respectively. This notable rate performance of GeTe-TiC (20%)-C and GeTe-TiC (30%)-C can be ascribed to the coexistence of the hybrid TiC and C matrix, acting as a buffering agent with high conductivity and minimizing the large volume expansion during cycling.

**Figure 7:**
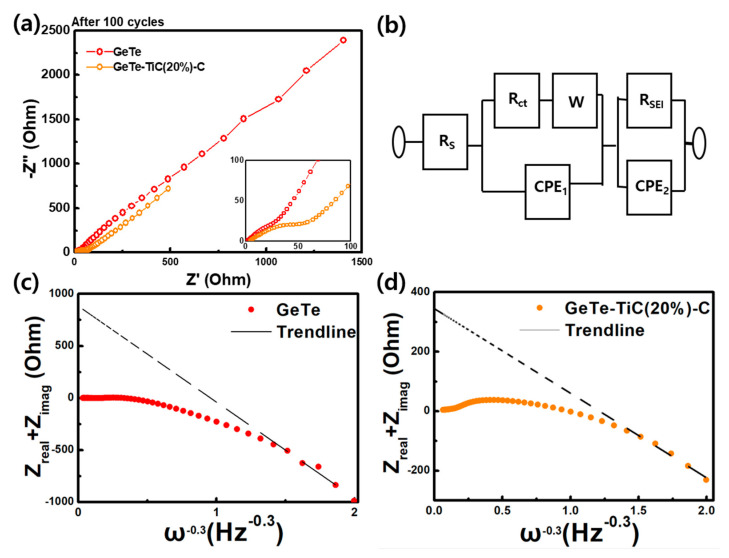
Nyquist plots for electrochemical impedance spectroscopy (EIS) spectra of GeTe and GeTe-TiC (20 %)-C electrodes (**a**) after 100 cycles, with (**b**) the corresponding equivalent circuit. (Z_real_ + Z_imag_) plots of GeTe electrode (**c**) and GeTe-TiC (20 %)-C electrode (**d**).

EIS measurements were performed after 100 cycles to analyze the relationship between the cycling performance and resistance, as displayed in Figure 7. Nyquist plots were obtained from GeTe and GeTe-TiC (20%)-C electrodes (Figure 7a), which includes semicircles in a high-frequency zone. A modified Randles circuit was selected as an equivalent circuit, which contains series resistance (R_s_), charge-transfer resistance (R_ct_), SEI resistance (R_SEI_), and a Warburg element (W) (Figure 7b). To improve the simulation’s goodness of fit, constant-phase elements (CPEs) were assigned to replace double-layer capacitors; the simulation results are summarized in Table 2. Even though the uncertainty in determining R_s_ (±1.0 Ω) and R_SEI_ (±58.1 Ω) was satisfactory, the simulated R_ct_ was highly unreliable due to its tremendous uncertainty (±145 kΩ). The primary criterion in EIS fitting was minimizing the difference between an observed pattern and a simulated one. As the total impedance of the equivalent circuit consists of nine independent parameters, there could be many solution sets satisfying the primary criterion [63,64]. By varying parameters within their uncertainties, the simulated patterns were nearly identical. For instance, unless the values of R_ct_ for the GeTe electrode exceeded 163 kΩ (upper limit), the best goodness of fitting could still be mathematically achieved via varying other parameters such as capacitances or the Warburg coefficient. By applying further constraints or considering secondary criteria, we could reduce the statistical uncertainty and increase the physical meaning of the refined parameters. Therefore, the low-frequency constraint suggested by Vo et al. was applied to refine R_ct_ [65]. In Figure 7c and d, the trend line of the sum of impedances (Z_real_ + Z_imag_) at low frequency had a negative slope and an X-intercept of 882 Ω, which are the sum of resistances (R_s_ + R_SEI_ + R_ct_). Since the simulated values of both R_s_ and R_SEI_ were dependable, the value of R_ct_ was estimated as 848 Ω for the GeTe electrode. Similarly, in the case of the GeTe-TiC (20%)-C electrode, the low-frequency constraint also proposed that the value of R_ct_ could be 278 Ω. These results indicate that the introduction of TiC and C did not considerably influence the electrical conductivity of the SEI layers, as the values of R_SEI_ for electrodes prepared from GeTe and GeTe-TiC (20 %)-C were nearly identical. Meanwhile, the overall R_ct_ was reduced three times by introducing TiC-C (from 848 Ω down to 278 Ω). In 2015, Allcorn and Mathiram also analyzed EIS spectra of FeSb-TiC-C composite electrodes and observed that all the composite electrodes initially had similar R_ct_ regardless of TiC content [53]. However, the R_ct_ values of the TiC-lesser electrodes drastically increased with cycling time, whereas those of the TiC-rich electrodes hardly changed. Since the charge-transfer kinetic strongly relied on the connectivity of the active phase, low R_ct_ was usually achieved for well-connected particles. As TiC alleviated the pulverization and supplied good connectivity, the R_ct_ values of the TiC-rich electrodes hardly increased with cycling time. In contrast, without TiC, the alloying-type electrodes usually suffered a great volume expansion and quickly lost their connectivity through operation [10]. Therefore, the R_ct_ of the GeTe electrode dramatically increased through cycling. Accordingly, after 100 cycles, the GeTe-TiC (20 %)-C electrode demonstrated much lower R_ct_ than the GeTe electrode because TiC-C well maintained the connectivity among GeTe particles. Having a small R_ct_ and a negligible R_SEI_ undoubtedly proved that the GeTe-TiC (20%)-C electrode was suitable for fast charging application, which was consistent with the rate characterization (Figure 6c,d). Therefore, based on the cyclic performance and impedance results, it is evident that the GeTe-TiC-C electrodes are viable alloy anodes for high-performance LIBs.

## 4. Conclusions

In this study, GeTe–TiC-C composites were synthesized via high-energy ball milling (HEBM). From the milled product, crystalline GeTe and TiC phases were dispersed in a carbon matrix. The prepared electrode displayed superior electrochemical performance compared with the GeTe electrode. GeTe displayed a low capacity retention of 58% after 100 cycles, whereas GeTe-TiC-C electrodes exhibited exceedingly high capacity retentions. The TiC crystallites with high conductivity helped in mitigating large volume changes in the amorphous carbon matrix as the TiC content increased, ensuring outstanding cyclic life. Specifically, GeTe-TiC (20%)-C and GeTe-TiC (30%)-C electrodes displayed excellent volumetric and specific capacities, corresponding to 1657 mAh cm^−3^ and 690 mAh g^−1^, 1281 mAh cm^−3^, and 557 mAh g^−1^, respectively, after 200 cycles. Moreover, at 10 A g^−1^, the GeTe-TiC (20%)-C and GeTe-TiC (30%)-C retained a high reversible capacity (78%–82%). Therefore, the outstanding electrochemical properties of the GeTe-TiC-C composite materials suggest a potential alternative to graphite for high-performance lithium-ion cells.

## Figures and Tables

**Table 1:** Electrochemical properties of GeTe and GeTe-TiC-C electrodes.

Sample	1st Charge Capacity (mAh g^−1^)	1st Discharge Capacity (mAh g^−1^)	Initial Coulombic Efficiency (%)	Capacity Retention at 100th Cycle (%)
GeTe	679	987	69	58
GeTe-TiC (10%)-C	655	778	84	98
GeTe-TiC (20%)-C	558	729	76	99
GeTe-TiC (30%)-C	508	722	70	99

**Table 2:** EIS data of GeTe and GeTe-TiC-C electrodes after 100 cycles without constraint verification.

Sample	R_s_ (Ω)	R_ct_ (Ω)	R_SEI_ (Ω)	α
GeTe	1.5 ± 1.0	15,771 ± 148,054	32.3 ± 58.1	0.7
GeTe-TiC (20%)-C	5.7 ± 1.8	618.3 ± 19,590	60.7 ± 32.9	0.7

## Supplementary Materials

The following are available online at https://www.mdpi.com/1996-1944/13/19/4222/s1, Figure S1: Discharge-charge curves of (a) GeTe, (b) GeTe-TiC (20%)-C and (c) GeTe-TiC (30%)-C electrodes, Table S1: Comparison on the electrochemical performances of electrodes with TiC content. For other data, they are adapted from the cited references.
