# Supplementary material for: GeTe-TiC-C Composite Anodes for Li-Ion Storage
Source: Materials (Basel). 2020 Sep 23;13(19):4222. doi: 10.3390/ma13194222 (PMC7579072; doi:10.3390/ma13194222)
Supplement: Supplementary file 1 [file materials-13-04222-s001.pdf]

materials

MDPI

Supplementary Materials

GeTe-TiC-C Composite Anodes for Li-Ion Storage

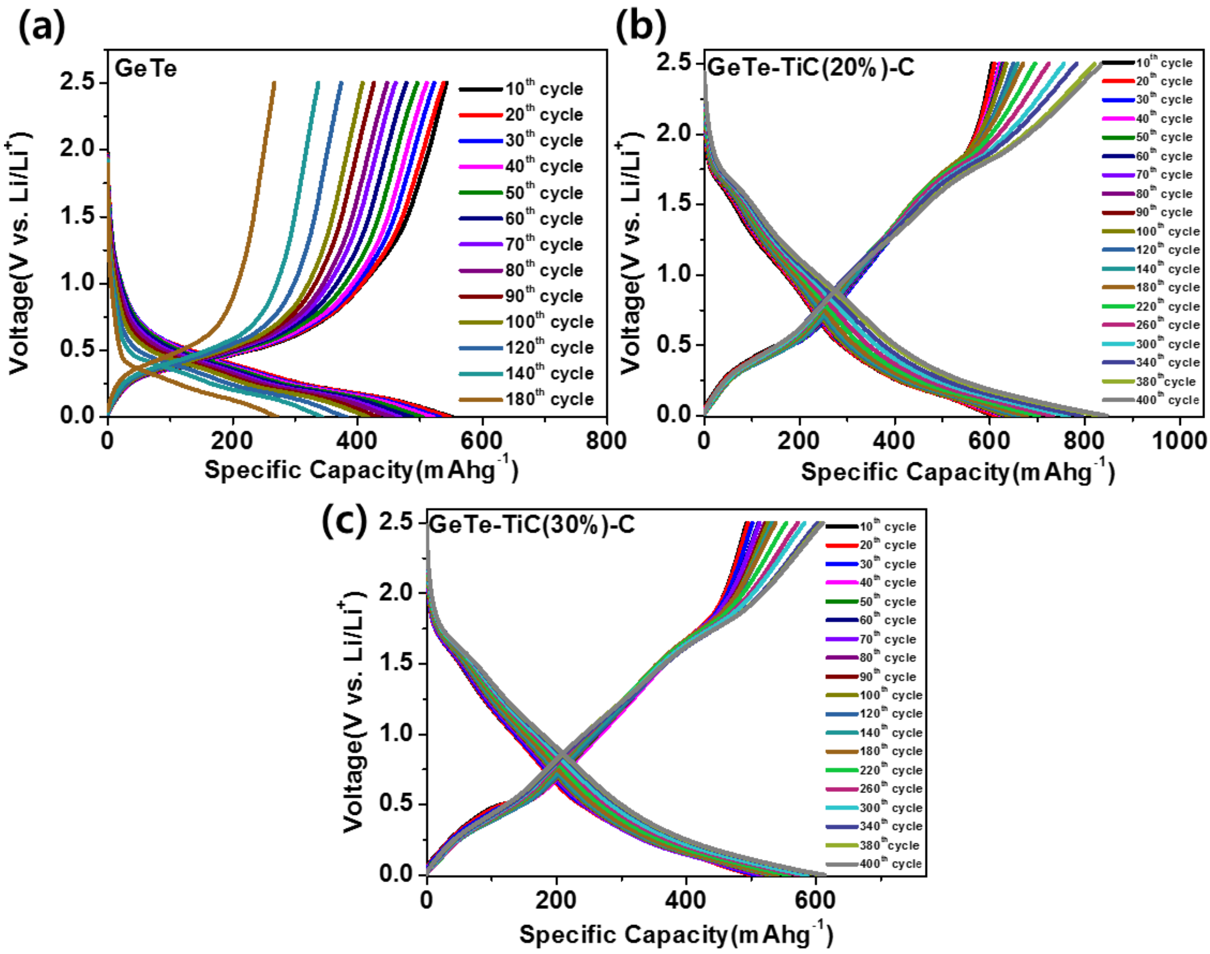

Figure S1. Discharge-charge curves of (**a**) GeTe, (**b**) GeTe-TiC (20%)-C and (**c**) GeTe-TiC (30%)-C electrodes.

Table S1. Comparison on the electrochemical performances of electrodes with TiC content. For other data, they are adapted from the cited references.

**Sample**	**1st Charge Capacity (mAh g^{-1})**	**Current Density (mA g^{-1})**	**Charge Capacity (mAh g^{-1})**	**Ref.**
Zn-TiC-C	402	100	~380 at 800th cycle	[1]
ZnTe@TiC (20%)-C	446	100	~530 at 300th cycle	[2]
SnSb-TiC-C	653	100	~430 at 100th cycle	[3]
Sn-TiC-C	390	100	~390 at 300th cycle	[4]
SnTe-TiC (30%)-C	419	100	~420 at 100th cycle	[5]
Sb_2Te_3-TiC (30%)-C	388	100	~330 at 600th cycle	[6]
$FeSn_2$-TiC	398	100	~380 at 100th cycle	[7]
GeTe-TiC (20%)-C	588	100	766 at 300th cycle<br>847 at 400th cycle	Our work
GeTe-TiC (30%)-C	508	100	588 at 300th cycle<br>614 at 400th cycle	

References

1. Kim, S.O.; Manthiram, A. High-performance Zn-TiC-C nanocomposite alloy anode with exceptional cycle life for lithium-ion batteries. *ACS Appl. Mater. Interfaces* **2015**, *7*, 14801–14807, doi:10.1021/acsami.5b03110.
2. Nguyen, Q.H.; Nguyen, Q.H.; So, S.; Hur, J. Efficient TiC-C hybrid conductive matrix for ZnTe anode in Lithium-ion storage. *Appl. Surf. Sci.* **2020**, *534*, 147679, doi:10.1016/j.apsusc.2020.147679.
3. Leibowitz, J.; Allcorn, E.; Manthiram, A. SnSb-TiC-C nanocomposite alloy anodes for lithium-ion batteries. *J. Power Sources* **2015**, *279*, 549–554, doi:10.1016/j.jpowsour.2015.01.055.
4. Yoon, S.; Manthiram, A. Nanoengineered Sn-TiC-C composite anode for lithium ion batteries. *J. Mater. Chem.* **2010**, *20*, 236–239, doi:10.1039/b919116j.
5. Son, S.Y.; Hur, J.; Kim, K.H.; Son, H.B.; Lee, S.G.; Kim, I.T. SnTe-TiC-C composites as high-performance anodes for Li-ion batteries. *J. Power Sources* **2017**, *365*, 372–379, doi:10.1016/j.jpowsour.2017.08.105.
6. Kim, H.; Kim, M.; Yoon, Y.H.; Nguyen, Q.H.; Kim, I.T.; Hur, J.; Lee, S.G. Sb_2Te_3-TiC-C nanocomposites for the high-performance anode in lithium-ion batteries. *Electrochim. Acta* **2019**, *293*, 8–18, doi:10.1016/j.electacta.2018.10.002.
7. Leibowitz, J.; Allcorn, E.; Manthiram, A. $FeSn_2$-TiC nanocomposite alloy anodes for lithium ion batteries. *J. Power Sources* **2015**, *295*, 125–130, doi:10.1016/j.jpowsour.2015.06.144.

© 2020 by the authors. Submitted for possible open access publication under the terms and conditions of the Creative Commons Attribution (CC BY) license (http://creativecommons.org/licenses/by/4.0/).
